# Supplementary material for: Anatomical factors associated with the lingual fracture pattern in sagittal split ramus osteotomy: A case-control study
Source: PLoS One. 2025 Aug 5;20(8):e0329542. doi: 10.1371/journal.pone.0329542 (PMC12324670; doi:10.1371/journal.pone.0329542)
Supplement: S1 Dataset — (PDF) [file pone.0329542.s001.pdf]

Groups	Age	Sex	Facial deformity	Fracture pattern	Third Molar	Canal-Cortical distance	Ramus thickness	Ramus length	Ramus height	Lingula-Notch distance	Lingula-Anterior border distance	Lingula-Inferior border distance	Lingula-Posterior border distance
01	18	01	03	03	02	04	04	28	44	16	16	28	12
01	18	02	03	03	02	05	05	36	52	15	20	37	16
01	18	01	03	03	02	04	04	27	46	15	16	31	12
01	18	02	03	03	02	04	04	29	56	19	17	38	12
01	19	01	03	03	02	04	04	30	38	12	16	25	14
01	20	02	03	03	02	04	04	34	57	21	17	36	18
01	20	01	03	03	02	04	04	22	40	14	13	27	09
01	20	02	03	03	02	04	04	29	53	21	14	32	14
01	21	02	03	03	02	03	03	31	47	14	16	33	15
01	21	02	03	03	02	05	05	30	51	15	16	36	14
01	21	02	03	03	02	07	07	31	49	15	18	35	14
01	21	01	02	03	02	04	04	26	39	14	14	25	12
01	21	02	03	03	02	03	03	31	47	16	17	30	14
01	21	02	03	03	01	04	04	27	49	18	14	31	13
01	21	02	03	04	01	04	04	30	52	21	16	31	15
01	22	01	02	03	02	02	02	27	37	14	15	22	12
01	22	01	02	03	02	03	03	25	42	13	12	29	13
01	22	01	03	03	02	04	04	34	50	20	21	30	14
01	22	01	03	03	01	03	03	24	44	15	13	29	11
01	23	02	03	03	02	03	03	26	51	19	12	33	14
01	23	01	03	04	02	03	03	29	39	11	15	27	14
01	23	02	03	03	02	03	03	25	38	12	12	27	13
01	24	02	03	03	02	04	04	31	55	16	16	39	15
01	24	02	03	03	02	03	03	32	55	18	15	37	17
01	25	02	03	04	02	04	04	30	48	14	16	34	14
01	25	02	03	03	02	05	05	30	49	13	17	36	13
01	25	01	03	03	02	03	03	28	46	18	13	29	15
01	26	02	03	04	02	04	04	27	49	17	12	33	15
01	26	01	02	03	02	04	04	28	44	13	13	32	15
01	26	01	02	03	02	04	04	28	47	15	14	32	14
01	26	01	03	03	02	04	04	25	45	16	15	29	11

01	26	01	03	03	02	03	03	28	44	14	15	30	13
01	26	01	03	03	02	03	03	29	43	14	17	29	12
01	27	02	03	04	02	06	06	33	47	14	19	33	13
01	27	02	03	04	02	06	06	31	50	15	15	35	16
01	27	02	03	04	02	03	03	31	40	13	18	28	13
01	27	02	03	03	02	05	05	27	50	19	13	31	14
01	27	02	03	03	02	05	05	27	48	16	14	31	13
01	27	02	03	04	02	03	03	32	55	21	15	34	17
01	27	02	03	03	02	03	03	31	38	11	18	27	13
01	27	02	03	03	02	08	08	28	49	17	14	32	14
01	27	02	02	04	01	03	03	25	39	13	14	27	11
01	28	01	02	03	02	05	05	29	45	14	17	31	12
01	28	01	02	03	02	05	05	31	46	14	18	32	14
01	28	02	03	03	02	05	05	31	47	19	17	28	13
01	28	01	02	03	02	04	04	26	45	15	15	31	11
01	28	02	03	03	02	05	05	29	50	13	16	37	13
01	29	01	03	03	02	04	04	30	47	14	17	32	13
01	29	01	03	03	01	04	04	28	40	15	15	25	13
01	29	01	03	03	01	02	02	29	43	15	15	28	14
01	30	01	03	04	02	03	03	27	42	13	17	29	10
01	30	01	02	03	02	04	04	27	41	14	18	27	10
01	30	02	02	03	02	04	04	30	36	13	16	23	14
01	31	01	02	04	01	05	05	28	43	16	15	27	13
01	33	02	03	03	02	06	06	13	55	21	17	34	-04
01	33	02	02	03	01	03	03	26	48	13	13	35	13
01	34	02	02	03	01	04	04	31	41	15	18	26	13
01	35	01	03	03	02	05	05	26	40	16	15	24	12
01	35	01	03	03	02	04	04	26	41	16	14	25	12
01	36	01	02	03	02	04	04	25	33	12	15	21	11
01	37	01	03	03	02	05	05	28	37	12	14	26	14
01	37	01	03	03	02	04	04	24	37	12	11	25	13
01	37	01	02	03	02	05	05	24	48	21	11	26	13
01	38	01	02	04	02	04	04	27	35	11	15	24	12
01	38	01	02	03	02	05	05	25	37	12	14	26	11

01	38	01	03	03	02	05	05	31	46	17	17	29	14
01	38	01	03	03	02	05	05	33	48	17	18	31	15
01	40	01	02	03	02	03	03	20	32	12	11	20	09
01	40	01	02	03	01	04	04	28	40	13	16	27	12
01	40	01	02	03	01	03	03	27	39	14	15	26	12
01	41	02	02	03	02	04	04	30	46	16	15	30	14
01	41	01	02	04	02	03	03	26	41	12	17	29	09
01	45	01	02	04	02	05	05	24	44	19	11	25	13
01	45	01	02	04	02	05	05	23	40	13	11	27	12
01	46	02	03	03	02	03	03	28	46	15	16	32	13
01	46	02	03	03	02	03	03	27	49	16	14	33	13
01	52	01	02	03	02	04	04	28	37	12	16	25	12
01	23	01	03	03	01	05	05	25	41	15	15	27	10
01	23	01	03	03	01	05	05	25	44	15	15	29	10
01	27	01	02	03	02	04	04	28	35	11	15	25	13
01	23	01	02	03	02	04	04	24	36	11	13	25	11
01	27	01	03	03	02	05	05	30	51	15	15	36	15
01	27	01	03	03	02	03	03	30	51	16	14	35	16
01	21	02	03	03	02	05	05	33	56	18	17	38	16
01	21	02	03	03	02	06	06	34	59	22	17	37	17
01	28	01	02	03	02	04	04	21	37	13	11	24	10
01	28	01	02	03	02	04	04	21	35	14	13	21	08
01	21	02	03	03	02	03	07	29	55	20	15	35	14
01	29	02	03	04	02	03	03	26	45	16	13	29	13
01	29	02	03	03	02	03	03	28	45	16	14	29	14
02	18	01	03	02	01	04	08	27	43	14	15	29	12
02	18	01	03	01	01	04	08	28	41	14	16	27	11
02	18	01	03	01	01	06	11	29	46	17	16	29	13
02	18	01	03	01	01	07	11	28	50	20	16	29	12
02	18	01	03	02	01	06	10	32	52	15	18	37	14
02	18	01	03	02	01	05	09	29	50	19	15	31	13
02	18	02	03	01	02	04	07	34	55	15	20	39	15
02	18	01	03	01	02	05	10	25	40	15	15	25	10
02	18	01	03	02	02	05	09	23	37	16	13	21	10

02	18	02	03	01	02	03	08	30	51	22	16	29	15
02	18	02	03	01	02	03	08	30	50	22	12	28	18
02	18	02	03	02	02	04	11	27	58	19	16	40	12
02	19	02	03	01	02	05	11	31	51	16	17	35	14
02	19	02	03	02	02	04	10	31	49	16	18	32	13
02	19	01	03	02	02	06	11	28	42	14	16	29	13
02	19	01	03	02	02	06	11	28	41	12	15	28	13
02	19	01	03	01	02	05	09	31	37	13	18	24	13
02	20	01	03	01	02	04	08	29	39	12	14	27	14
02	20	01	03	01	02	03	08	29	37	15	16	23	13
02	20	02	03	01	02	03	09	28	51	18	15	32	13
02	20	02	03	01	02	04	08	26	53	18	15	34	11
02	20	01	03	02	02	04	08	23	40	13	14	28	09
02	20	02	03	02	02	03	07	36	55	21	17	34	18
02	20	02	03	01	02	05	08	30	50	17	16	33	14
02	21	01	02	01	02	05	08	25	41	13	14	28	11
02	22	01	03	01	02	04	08	32	49	18	18	32	14
02	22	01	03	02	01	04	09	28	45	16	16	30	12
02	23	02	03	01	02	04	08	28	55	19	14	36	13
02	23	02	03	01	02	04	07	29	53	19	15	34	14
02	23	01	03	01	02	03	07	31	40	11	18	29	14
02	24	01	03	01	02	05	07	29	46	22	15	25	14
02	24	01	03	01	02	04	07	30	47	18	16	29	14
02	24	01	02	02	02	06	10	26	42	11	16	31	10
02	24	01	02	01	02	05	09	29	44	10	17	34	11
02	25	01	03	01	02	03	07	27	49	22	13	27	15
02	26	02	03	01	02	05	11	33	51	15	17	36	16
02	26	02	03	01	02	05	10	34	51	17	19	35	15
02	26	01	03	01	02	03	07	29	45	13	15	32	14
02	26	01	03	02	02	02	07	25	43	15	14	28	11
02	26	01	03	01	02	05	09	29	42	14	16	29	12
02	26	02	03	01	02	04	07	26	53	16	13	36	13
02	27	02	03	01	02	03	08	24	40	11	12	30	12
02	27	02	03	01	01	04	08	23	41	13	11	28	12

02	27	01	03	01	02	04	09	28	36	12	16	24	11
02	27	01	03	01	02	05	10	29	37	09	18	28	12
02	27	02	02	02	02	02	06	26	38	11	13	28	12
02	27	02	03	02	02	09	14	29	51	20	15	31	15
02	27	02	03	01	02	03	08	31	53	23	17	30	15
02	28	02	03	01	02	04	10	31	47	19	17	28	13
02	28	01	03	02	02	04	09	27	45	16	15	28	11
02	28	01	03	01	02	03	08	26	45	17	14	28	12
02	28	01	03	02	02	06	09	28	37	11	13	26	15
02	28	01	03	01	02	06	08	27	37	09	14	28	14
02	28	01	01	02	02	04	07	26	40	14	13	26	13
02	28	01	01	01	02	03	08	29	43	13	15	30	14
02	28	01	02	01	01	05	09	28	44	15	15	29	13
02	28	02	03	01	01	06	09	30	50	16	15	34	15
02	29	01	03	02	02	04	09	28	47	15	16	32	12
02	30	02	02	01	02	04	09	30	43	15	15	28	16
02	30	01	02	01	02	04	08	28	42	13	17	29	11
02	30	01	03	01	02	03	09	26	43	14	15	29	11
02	31	02	03	01	02	05	10	34	56	20	18	36	17
02	31	02	03	01	02	05	09	37	58	22	17	36	20
02	31	01	02	01	01	07	11	29	42	15	15	26	14
02	31	01	02	01	02	05	10	31	39	15	16	24	15
02	31	01	02	01	02	04	10	31	33	14	17	18	13
02	33	01	03	02	02	05	08	28	45	14	14	31	14
02	33	01	03	02	02	05	09	28	46	14	14	32	14
02	33	02	03	01	02	07	12	30	54	20	17	34	12
02	33	02	02	01	02	04	08	27	46	16	14	30	13
02	34	02	02	01	01	04	07	29	40	14	16	26	13
02	36	01	02	01	02	03	07	27	38	14	13	24	13
02	37	01	02	01	02	05	09	25	47	20	12	27	13
02	40	01	02	02	02	02	08	19	32	13	10	19	09
02	41	01	02	01	02	05	10	28	43	13	16	30	12
02	41	02	02	01	02	05	09	31	45	15	16	31	15
02	52	01	02	01	02	05	09	29	39	14	16	25	13

02	26	01	02	01	02	11	06	31	44	15	17	29	14
02	26	01	02	02	02	05	10	31	44	14	16	30	15
02	27	01	02	01	02	04	07	28	35	11	15	25	13
02	24	02	03	01	02	05	09	36	39	11	20	29	16
02	24	02	03	01	02	04	09	32	40	10	17	30	15
02	37	01	03	02	02	03	10	37	42	12	19	29	18
02	37	01	03	01	02	05	11	36	39	13	19	27	17
02	18	02	02	01	02	03	09	35	48	17	20	31	15
02	18	02	02	02	02	04	10	35	50	18	21	33	13
02	23	01	02	01	02	04	10	23	38	14	13	24	11
02	39	01	02	01	02	06	12	24	46	16	13	31	11
02	39	01	02	01	02	06	12	24	46	14	14	32	11
02	21	02	03	01	02	03	08	31	53	21	16	32	14
