# Supplementary material for: Anatomical factors associated with the lingual fracture pattern in sagittal split ramus osteotomy: A case-control study
Source: PLoS One. 2025 Aug 5;20(8):e0329542. doi: 10.1371/journal.pone.0329542 (PMC12324670; doi:10.1371/journal.pone.0329542)
Supplement: Attachment — Submitted filename: PONE-D-25-23674 reviewed.pdf [file pone.0329542.s006.pdf]

PLOS ONE

Anatomical factors associated with the lingual fracture pattern in sagittal ramus osteotomy: a case-control study

--Manuscript Draft--

**Manuscript Number:**	PONE-D-25-23674
**Article Type:**	Research Article
**Full Title:**	Anatomical factors associated with the lingual fracture pattern in sagittal ramus osteotomy: a case-control study
**Short Title:**	Anatomical Factors Associated with the Lingual Fracture Pattern in Sagittal Osteotomy
**Corresponding Author:**	Carla Cecília Lira Pereira de Castro<br>UPE: Universidade de Pernambuco<br>Recife, Pernambuco BRAZIL
**Keywords:**	Orthognathic surgery; Sagittal split ramus osteotomy; Unfavorable fractures; Computed tomography.
**Abstract:**	Unfavorable fractures are among the most challenging complications in sagittal split ramus osteotomy (SSRO), potentially increasing surgical morbidity and compromising postoperative outcomes. The preoperative identification of anatomical risk factors through imaging may enhance surgical planning and prevent such events. This study aims to investigate the anatomical factors associated with lingual fracture patterns in SSRO using multislice computed tomography. This retrospective case-control study included 180 mandibular rami from patients who underwent SSRO at a Clinical Research Center for Oral and Maxillofacial Surgery. Fractures were classified according to Plooij (2009), with Types 3 and 4 grouped as cases, and Types 1 and 2 as controls. Linear measurements of mandibular ramus thickness, the distance between the mandibular canal and the buccal cortical bone, as well as parameters related to the mandibular lingula were analyzed. The case group showed significantly thinner mandibular bone (p<0.001) and a shorter canal-to-cortical distance (p=0.013), suggesting a direct association between these anatomical variables and unfavorable fracture patterns. Bonferroni post hoc analysis revealed no significant difference between Type 3 and Type 4 fracture patterns (p=1.000), supporting their grouping into a single analytical category. The presence of third molars was not significantly associated with fracture patterns (p>0.05). These findings highlight the importance of anatomical parameters in predicting the risk of unfavorable fractures. Specifically, reduced bone thickness and proximity of the mandibular canal play a crucial role in the occurrence of unfavorable SSRO fractures. Preoperative evaluation using computed tomography is essential to optimize surgical planning and minimize complications.
**Order of Authors:**	Thalles Moreira Suassuna
	Elenisa Glaucia Ferreira dos Santos
	Sérgio Murilo Cordeiro de Melo Filho
	Carla Cecília Lira Pereira de Castro
	Tatiane Fonseca Faro
	Fernanda Souto Maior dos Santos Araújo
	Allan Vinícius Martins-de-Barros
	Fábio Andrey da Costa Araújo
**Additional Information:**	
**Question**	**Response**
**Financial Disclosure**<br><br>Enter a financial disclosure statement that describes the sources of funding for the	The author(s) received no specific funding for this work.

Powered by Editorial Manager® and ProduXion Manager® from Aries Systems Corporation

work included in this submission. Review the submission guidelines for detailed requirements. View published research articles from *PLOS ONE* for specific examples.<br><br>This statement is required for submission and **will appear in the published article** if the submission is accepted. Please make sure it is accurate.<br><br>**Funded studies**<br>Enter a statement with the following details:<br>• Initials of the authors who received each award<br>• Grant numbers awarded to each author<br>• The full name of each funder<br>• URL of each funder website<br>• Did the sponsors or funders play any role in the study design, data collection and analysis, decision to publish, or preparation of the manuscript?<br><br>Did you receive funding for this work?	
**Competing Interests**<br><br>Use the instructions below to enter a competing interest statement for this submission. On behalf of all authors, disclose any competing interests that could be perceived to bias this work—acknowledging all financial support and any other relevant financial or non-financial competing interests.<br><br>This statement is required for submission and **will appear in the published article** if the submission is accepted. Please make sure it is accurate and that any funding sources listed in your Funding Information later in the submission form are also declared in your Financial Disclosure statement.<br><br>View published research articles from *PLOS ONE* for specific examples.	The authors have declared that no competing interests exist.

Powered by Editorial Manager® and ProduXion Manager® from Aries Systems Corporation

**NO authors have competing interests**<br><br>Enter: *The authors have declared that no competing interests exist.*<br><br>**Authors with competing interests**<br><br>Enter competing interest details beginning with this statement:<br><br>*I have read the journal's policy and the authors of this manuscript have the following competing interests: [insert competing interests here]*<br><br>* typeset	
**Ethics Statement**<br><br>Enter an ethics statement for this submission. This statement is required if the study involved:<br><br>• Human participants<br>• Human specimens or tissue<br>• Vertebrate animals or cephalopods<br>• Vertebrate embryos or tissues<br>• Field research<br><br>Write "N/A" if the submission does not require an ethics statement.<br><br>General guidance is provided below. Consult the submission guidelines for detailed instructions. **Make sure that all information entered here is included in the Methods section of the manuscript.**	This is a retrospective case-control study conducted using secondary data at the Research Center of the Oswaldo Cruz University Hospital, in Recife, Pernambuco, Brazil, from December 2024 to January 2025. The study was approved by the Research Ethics Committee of the University of Pernambuco (Approval No. 6,570,319; CAAE No. 74613823.2.0000.5207). No participants under the age of 18 were included in this study. All participants were informed about the risks and benefits and signed an Informed Consent Form authorizing the use of tomographic images and the information contained in their medical records.

Powered by Editorial Manager® and ProduXion Manager® from Aries Systems Corporation

**Format for specific study types**<br><br>**Human Subject Research (involving human participants and/or tissue)**<br>• Give the name of the institutional review board or ethics committee that approved the study<br>• Include the approval number and/or a statement indicating approval of this research<br>• Indicate the form of consent obtained (written/oral) or the reason that consent was not obtained (e.g. the data were analyzed anonymously)<br><br>**Animal Research (involving vertebrate animals, embryos or tissues)**<br>• Provide the name of the Institutional Animal Care and Use Committee (IACUC) or other relevant ethics board that reviewed the study protocol, and indicate whether they approved this research or granted a formal waiver of ethical approval<br>• Include an approval number if one was obtained<br>• If the study involved *non-human primates*, add *additional details* about animal welfare and steps taken to ameliorate suffering<br>• If anesthesia, euthanasia, or any kind of animal sacrifice is part of the study, include briefly which substances and/or methods were applied<br><br>**Field Research**<br><br>Include the following details if this study involves the collection of plant, animal, or other materials from a natural setting:<br>• Field permit number<br>• Name of the institution or relevant body that granted permission	
**Data Availability**<br><br>Authors are required to make all data underlying the findings described fully available, without restriction, and from the time of publication. PLOS allows rare exceptions to address legal and ethical concerns. See the PLOS Data Policy and FAQ for detailed information.	Yes - all data are fully available without restriction

A Data Availability Statement describing where the data can be found is required at submission. Your answers to this question constitute the Data Availability Statement and **will be published in the article**, if accepted.<br><br>**Important:** Stating 'data available on request from the author' is not sufficient. If your data are only available upon request, select 'No' for the first question and explain your exceptional situation in the text box.<br><br>Do the authors confirm that all data underlying the findings described in their manuscript are fully available without restriction?	
**Describe where the data may be found in full sentences. If you are copying our sample text, replace any instances of XXX with the appropriate details.**<br><br>• If the data are **held or will be held in a public repository**, include URLs, accession numbers or DOIs. If this information will only be available after acceptance, indicate this by ticking the box below. For example: *All XXX files are available from the XXX database (accession number(s) XXX, XXX.*).<br>• If the data are all contained **within the manuscript and/or Supporting Information files**, enter the following: *All relevant data are within the manuscript and its Supporting Information files.*<br>• If neither of these applies but you are able to provide **details of access elsewhere**, with or without limitations, please do so. For example:<br><br>*Data cannot be shared publicly because of [XXX]. Data are available from the XXX Institutional Data Access / Ethics Committee (contact via XXX) for researchers who meet the criteria for access to confidential data.*<br><br>*The data underlying the results presented in the study are available from (include the name of the third party*	All relevant data are within the manuscript and its Supporting Information files.

and contact information or URL).<br>• This text is appropriate if the data are owned by a third party and authors do not have permission to share the data.<br><br>* typeset	
Additional data availability information:	Tick here if the URLs/accession numbers/DOIs will be available only after acceptance of the manuscript for publication so that we can ensure their inclusion before publication.

Powered by Editorial Manager® and ProduXion Manager® from Aries Systems Corporation

Manuscript

Click here to access/download;Manuscript;renamed_791f3.docx

Anatomical Factors Associated with the Lingual Fracture Pattern in Sagittal Split Ramus Osteotomy: A Case-Control Study

Running Title:

Anatomical Factors Associated with the Lingual Fracture Pattern in Sagittal Osteotomy

Authors:

Thalles Moreira Suassuna[1], Elenisa Glaucia Ferreira dos Santos[1], Sérgio Murilo Cordeiro de Melo Filho[2], Carla Cecília Lira Pereira de Castro[2], Tatiane Fonseca Faro[3], Fernanda Souto Maior dos Santos Araújo[4], Allan Vinícius Martins-de-Barros[1,3], Fábio Andrey da Costa Araújo[2,3]

[1] University of Pernambuco, Postgraduate Program in Dentistry, Recife, Brazil

[2] Department of Oral and Maxillofacial Surgery, School of Dentistry, University of Pernambuco, Recife, Brazil

[3] Hospital dos Servidores do Estado de Pernambuco, Recife, Brazil

[4] School of Dentistry, University of Pernambuco, Recife, Brazil

Corresponding Author:

E-mail: fabio.andrey@upe.br

¶ These authors contributed equally to this work.

Abstract:

Unfavorable fractures are among the most challenging complications in sagittal split ramus osteotomy (SSRO), potentially increasing surgical morbidity and compromising postoperative outcomes. The preoperative identification of anatomical risk factors through imaging can enhance surgical planning and prevent such events. This study aims to investigate the anatomical factors associated with lingual fracture patterns in SSRO using multislice computed tomography. This retrospective case-control study included 180 mandibular rami from patients who underwent SSRO at a Clinical Research Center for Oral and Maxillofacial Surgery. Fractures were classified according to Plooij (2009), with Types 3 and 4 grouped as cases and Types 1 and 2 as controls. Linear measurements of mandibular ramus thickness, the distance between the mandibular canal and the buccal cortical bone, as well as parameters related to the mandibular lingula were analyzed. The case group showed significantly thinner mandibular bone ($p<0.001$) and a shorter canal-cortical distance ($p=0.013$), suggesting a direct association between these anatomical variables and unfavorable fracture patterns. Bonferroni post hoc analysis revealed no significant difference between fracture patterns Type 3 and Type 4 ($p=1.000$), supporting their grouping in a single analytical category. The presence of third molars was not significantly associated with fracture patterns ($p>0.05$). These results underscore the importance of anatomical parameters in predicting the risk of unfavorable fractures. Specifically, reduced bone thickness and proximity of the mandibular canal play a crucial role in the occurrence of unfavorable

SSRO fractures. Preoperative evaluation using computed tomography is essential to optimize surgical planning and minimize complications.

Keywords: Orthognathic surgery; Sagittal split ramus osteotomy; Unfavorable fractures; Computed tomography.

Introduction

Sagittal split ramus osteotomy (SSRO) is widely used in orthognathic surgery to correct dentofacial deformities such as prognathism, retrognathism, and mandibular asymmetries [1-4]. Initially described by Trauner and Obwegeser in 1957 and later modified by Dal Pont, the technique has been refined over the years, but the splitting step remains a challenging aspect of orthognathic surgery [5,6]. SSRO still presents some limitations, including difficulties in controlling the fracture pattern on the lingual surface, which may lead to unfavorable fractures (UF).

Plooij et al. (2009) classified mandibular fracture patterns during the bone split in SSRO into four types: Type 1 is a vertical fracture extending toward the inferior border of the ramus; Type 2 is a horizontal fracture directed toward the posterior border of the ramus; Type 3 is a vertical fracture passing through the mandibular canal toward the inferior border; and Type 4 is an unfavorable fracture involving the coronoid process and/or subcondylar region. The last two types may represent up to 20% of cases and are classified as non-conventional fracture patterns, thus increasing the risk of complications during orthognathic surgery [7-10].

The main complications associated with these fracture patterns include increased surgical trauma, infection, temporomandibular joint complications, and bone non-union

[11-13], making orthognathic surgery more challenging and associated with higher morbidity for the patient.

Studies suggest that anatomical factors may influence the risk of UF, such as the thickness of the cortical bone, fusion between buccal and lingual cortices, and the presence of third molars [10,14-16]. Jiang (2021) demonstrated that a shorter distance between the mandibular canal and the buccal cortex is associated with a higher risk of unfavorable fracture.

Understanding the anatomical characteristics that influence the fracture path—especially in the lingual region of the mandible—may contribute to more predictable and individualized UF risk assessment, reduced complications, and improved SSRO technique. Therefore, the aim of this study was to evaluate mandibular anatomical factors that may influence the fracture pattern on the lingual aspect of the mandibular ramus during SSRO.

Methodology

Study design and ethical considerations

This is a retrospective case-control study conducted at the Clinical Research Center in Oral and Maxillofacial Surgery and Traumatology of the Oswaldo Cruz University Hospital, in Recife, Brazil, from December 2024 to January 2025. The study was approved by the Research Ethics Committee of the University of Pernambuco (Approval No. 6,570,319; CAAE No. 74613823.2.0000.5207), and conducted in accordance with the Strengthening the Reporting of Observational Studies in Epidemiology (STROBE) guidelines for case-control studies. All patients were informed about the risks and benefits of the study and signed an Informed Consent

Form for the use of their tomographic images and the information contained in their medical records.

Study population and eligibility criteria

The study population consisted of individuals who underwent SSRO for correction of dentofacial deformities at a single hospital center in Northeast Brazil. All surgical procedures were performed by the same experienced oral and maxillofacial surgical team using the SSRO technique described by Dal Pont and modified by Hunsuck and Epker, followed by hybrid osteosynthesis using titanium plates and screws.

Eligible participants were men and women over 18 years old who had undergone surgical treatment for dentofacial deformities and had both pre- and postoperative tomographic images archived at the institution. Individuals with a history of facial trauma, reoperations, or no available postoperative CT imaging within 30 days of surgery were excluded.

Each mandibular ramus was considered an independent unit of analysis[illegible] Mandibular rami were divided into two groups based on postoperative CT scan analysis of the lingual split pattern in SSRO. Fractures were classified according to Plooij et al. (2009):

- Type 1: Vertical fracture extending toward the inferior border (True Hunsuck)
- Type 2: Horizontal fracture directed toward the posterior border of the ramus
- Type 3: Vertical fracture line through the mandibular canal toward the inferior border
- Type 4: Other or unfavorable fracture patterns

The **case group** included mandibular rami with non-conventional or unfavorable fracture patterns (Types 3 and 4), while the **control group** included rami with conventional fracture patterns (Types 1 and 2) (Fig. 1).

Figure 1: Comparison between Plooij's (2009) classification and the patterns observed in this study based on 3D CT reconstructions.

CT Image Analysis

To evaluate mandibular anatomical parameters, pre and immediate postoperative facial computed tomography (CT) scans were performed using a multislice scanner, with 0.7 mm slices, 0.4 mm voxel resolution, and parameters of 120 kV and 100 mAs/slice, saved in DICOM format.

Preoperative images were retrospectively analyzed using Dolphin Imaging® software (version 11.95). A single examiner, blinded to group allocation, conducted all image evaluations. The assessed parameters included linear measurements of the mandibular ramus and the presence or absence of third molars in the mandibular arch. Linear measurements were obtained using the "Measure" tool in 2D line mode in the aforementioned software.

The thickness of the mandibular ramus at the level of the lingula was measured on coronal slices, while the distance between the buccal cortex and the mandibular canal, the distance between the sigmoid notch and the inferior border of the mandibular ramus, the distance from the posterior to the anterior border of the mandibular ramus, and the linear distances from the lingula to the anterior, inferior, and posterior borders of the ramus were obtained from 3D CT reconstructions. Additionally, the presence or absence of third molars was assessed using the panoramic reconstruction view (Fig. 2).

Figure 2: Linear measurement from the buccal cortex of the mandibular ramus to the outer cortex of the mandibular canal and the ramus thickness at the level of the mandibular lingula (ML). (A) Linear distance from the ML to the sigmoid notch (B) Distance from the ML to the anterior border of the mandibular ramus (C) Distance from the ML to the inferior border of the mandibular ramus (D) Distance from the ML to the posterior border of the mandibular ramus (E) Ramus height (F) Ramus length

Postoperative CT scans were analyzed using RadiAnt DICOM Viewer® (v25.1). The 3D reconstructions were generated from axial slices through selective segmentation of the mandible to evaluate the lingual fracture pattern of the mandibular ramus (Fig. 1).

Database Construction and Statistical Analysis

A database was created using SPSS® software (Statistical Package for the Social Sciences), version 20.0, where descriptive and inferential statistical analyses were performed. Descriptive analysis included measures of central tendency and dispersion for quantitative variables, and absolute and relative frequencies for categorical variables.

Inferential statistical analysis was conducted to assess possible associations between SSRO fracture patterns and predictive variables. For quantitative predictors, the Kolmogorov–Smirnov normality test was performed, and since data followed a normal distribution, group comparisons were made using the independent samples t-test. For categorical predictors, the chi-square test with Bonferroni correction was applied. A significance level of 95% was adopted for all analyses.

Results

Initially, 390 SSRO cases were evaluated through tomographic records from the institution's database. Of these, 177 were excluded due to the unavailability of immediate postoperative CT scans (within 30 days of surgery), and 213 were considered eligible for the study. After reviewing the tomographic images, 33 mandibular rami were excluded—5 due to a history of facial trauma and 28 due to previous reoperations. The final sample consisted of 180 mandibular rami, equally distributed between the case group (n = 90) with non-conventional fracture patterns (Types 3 and 4) and the control group (n = 90) with conventional fracture patterns (Types 1 and 2) (Fig. 3).

Figure 3: Flowchart of the sample selection process.

The demographic and clinical characteristics of the sample are shown in Table 1. In the case group, 50 (47.2%) participants were female and 40 (54.1%) were male. In the control group, 56 (52.8%) were female and 34 (45.9%) male. The participants' ages ranged from 18 to 52 years, with a mean age of 27 (± 7.4) years. Regarding the type of deformity, 2 (1.1%) participants were classified as Class I, 56 (31.1%) as Class II, and 122 (67.8%) as Class III. Among the 25 (13.9%) participants who presented with third molars preoperatively, 13 (52.0%) were in the case group. No statistically significant differences were observed between the case and control groups regarding demographic data, third molar presence, or type of deformity ($p > 0.05$) (Table 1).

Table 1. **Distribution of 90 SSRO cases in each group according to sex, age, deformity type, and presence of third molars.**

**Variables**	**Case (n=90)**	**Control (n=90)**	**p-value**
**Sex - Female**	50 (47.2%)	56 (52.8%)	0.363
**Sex - Male**	40 (54.1%)	34 (45.9%)	

**Age (mean ± SD)**	27 ± 7.4	28.3 ± 7.4	0.25
**Age ≤ 27 years**	45 (50.0%)	42 (47.2%)	0.361
**Age > 27 years**	45 (50.0%)	48 (54.2%)	
**Class I**	0 (0.0%)	2 (0.9%)	
**Class II**	30 (53.6%)	26 (46.4%)	0.314
**Class III**	60 (49.2%)	62 (50.8%)	
**3rd Molar Present**	13 (52.0%)	12 (48.0%)	
**3rd Molar Absent**	77 (49.7%)	78 (50.3%)	0.829

***p < 0.05**

The evaluation of mandibular anatomical factors aimed to identify potential associations with fracture patterns, and the results are presented in Table 2. The findings show that both the canal-cortical distance and mandibular ramus thickness were significantly lower in the case group. Conversely, the total length and height of the ramus did not differ significantly between groups ($p = 0.063$; $p = 0.987$). Distances between the mandibular lingula (ML) and the sigmoid notch, as well as the anterior, inferior, and posterior ramus borders, showed no significant differences between conventional and non-conventional fracture groups.

Table 2. **Analysis of mandibular anatomical factors associated with fracture pattern in the groups.**

**Variables**	**Case (n=90)**	**Control (n=90)**	**p-value**
**Canal-cortical distance (mm)**	3.9 ± 1.0	4.4 ± 1.3	0.013*
**Ramus thickness (mm)**	4.0 ± 1.1	8.7 ± 1.4	0.000*
**Ramus length (mm)**	27.8 ± 3.4	28.8 ± 3.4	0.063
**Ramus height (mm)**	44.8 ± 6.1	44.8 ± 5.9	0.987

**ML–sigmoid notch (mm)**	15.1 ± 2.8	15.3 ± 3.2	0.669
**ML–anterior border (mm)**	15.0 ± 2.0	15.5 ± 2.0	0.085
**ML–inferior border (mm)**	29.6 ± 4.3	29.4 ± 4.0	0.739
**ML–posterior border (mm)**	12.8 ± 2.5	13.2 ± 1.9	0.219

***p < 0.05**

ML: Mandibular Lingula

Bonferroni post hoc analysis was used to compare differences in mandibular ramus thickness between SSRO fracture patterns according to Plooij's (2009) classification. The multiple comparison revealed no statistically significant difference in ramus thickness between fracture types 3 and 4 (p = 1.000), suggesting both patterns share similar biomechanical characteristics (Table 3).

Table 3. Multiple comparison between fracture patterns for mandibular ramus thickness using Bonferroni post hoc test.

Compared Patterns	Mean Difference (mm)	95% CI Lower	95% CI Upper	p-value	Significant
**Type 1 vs. Type 2**	-0.425	-1.035	0.185	0.959	No
**Type 1 vs. Type 3**	4.569	4.003	5.135	0.000	Yes
**Type 1 vs. Type 4**	4.781	3.948	5.614	0.000	Yes

**Type 2 vs. Type 3**	4.994	4.418	5.569	0.000	Yes
**Type 2 vs. Type 4**	5.206	4.382	6.029	0.000	Yes
**Type 3 vs. Type 4**	0.212	-0.813	1.236	1.000	No

*Bonferroni post hoc testing adjusts the p-value for multiple comparisons to reduce the risk of false positives. A p-value greater than 0.05 indicates no significant difference between groups.

Discussion

The occurrence of unfavorable fractures during sagittal split ramus osteotomy (SSRO) has been widely investigated in the literature with the aim of identifying anatomical risk factors that may predispose to this type of complication. The results of this study are consistent with previous findings indicating that bone thickness and the distance between the mandibular canal and buccal cortex are significantly associated with non-conventional fracture patterns [16,19].

Mandibular ramus thickness was one of the main variables associated with fracture pattern. Our data demonstrated that reduced ramus thickness was significantly more frequent in the case group, a finding consistent with previous studies showing that thinner anatomical structures along the osteotomy pathway are associated with a higher predisposition to unfavorable fractures [16,19]. Telha et al. (2023) observed that a reduction in cancellous bone thickness, as well as decreased distance between the mandibular canal and the buccal cortex in the region of the first molar, led to unfavorable fractures during surgery.

Another relevant anatomical factor identified in this study was the distance between the mandibular canal and the buccal cortex. Proximity of the mandibular canal to the buccal cortex has been recognized as a risk factor for unfavorable fractures, as this anatomical configuration reduces the amount of cancellous bone available to dissipate the forces applied during bone splitting [16,20]. In our study, the average distance in patients with non-conventional fracture patterns was 3.9 mm, consistent with the findings of Jiang et al. (2021), who reported that a distance of less than 4 mm between the mandibular canal and buccal cortex was significantly associated with unfavorable fracture risk during SSRO.

Although the distance from the mandibular lingula (ML) to the sigmoid notch and posterior ramus border did not show statistically significant associations with fracture pattern in our study, Smith et al. (1991) concluded that performing horizontal osteotomies too high and too posterior relative to the ML significantly increases the risk of unfavorable fractures due to cortical fusion and lack of cancellous bone to absorb the splitting forces. Supporting this finding, De Souza Fernandes (2013) concluded that the horizontal osteotomy should not be performed more than 4 mm above the X-line proposed in his study, which extends from the most posterior point of the anterior border of the mandibular ramus to the posterior border, parallel to the mandibular base.

Significant differences were observed between Type 1 and Types 3 ($p = 0.000$) and 4 ($p = 0.000$), as well as between Type 2 and Types 3 ($p = 0.000$) and 4 ($p = 0.000$). The absence of a statistically significant difference between Types 3 and 4 justifies the inclusion of both patterns in the same analytical group (case group), based on the similarity of findings (Table 3). This similarity supports the grouping of these patterns into a single category for statistical analysis.

We thus opted to classify fracture patterns into two groups according to their potential clinical consequences, recognizing that the less conventional and more undesirable patterns correspond to Type 4 fractures (Plooij, 2009). The rationale for distinguishing between conventional and non-conventional fractures lies in the fact that surgical experience and anatomical analysis more reliably predict the occurrence of Type 1 and 2 fracture patterns after SSRO. Moreover, mandibular rami classified as Types 3 and 4 showed similar biomechanical characteristics in terms of ramus thickness, which warrants further investigation into possible clinical repercussions, such as neurosensory outcomes in patients from each group.

The presence of third molars was not significantly associated with fracture pattern, consistent with the findings of Camargo et al. (2016), who did not identify this variable as an independent risk factor for unfavorable fractures. This contrasts with studies suggesting that the presence of third molars increases the incidence of fractures [20], highlighting the need for further prospective studies to clarify this issue.

Our findings reinforce the importance of detailed preoperative assessment of mandibular anatomy to identify patients at higher risk for unfavorable fractures. The use of multislice computed tomography is essential for accurately measuring anatomical variables associated with this complication, enabling safer and more individualized surgical planning [21].

The results of this study emphasize the importance of careful evaluation of mandibular anatomical factors in preventing unfavorable fractures during SSRO. Preoperative identification of at-risk patients can contribute to the development of individualized surgical strategies, minimizing complications and improving clinical outcomes.

Supporting information

Author Contributions

Conceptualization: Fábio Andrey da Costa Araújo, Allan Vinícius Martins de-Barros, Tatiane Fonseca Faro.

Data curation: Thalles Moreira Suassuna, Fernanda Souto Maior dos Santos Araujo, Tatiane Fonseca Faro, Fabio Andrey da Costa Araujo.

Formal analysis: Fábio Andrey da Costa Araújo, Elenisa Glaucia Ferreira dos Santos, Sérgio Murilo Cordeiro de Melo Filho.

Investigation: Thalles Moreira Suassuna, Elenisa Glaucia Ferreira dos Santos, Sérgio Murilo Cordeiro de Melo Filho, Carla Cecília Lira Pereira de Castro.

Project administration: Fábio Andrey da Costa Araújo.

Supervision: Allan Vinícius Martins de-Barros, Tatiane Fonseca Faro.

Writing– original draft: Thalles Moreira Suassuna, Sérgio Murilo Cordeiro de Melo Filho, Carla Cecília Lira Pereira de Castro.

Writing– review & editing: Fernanda Souto Maior dos Santos Araujo, Allan Vinícius Martins de-Barros, Fábio Andrey da Costa Araújo.

References

1. Obgeweser HL. Orthognathic Surgery and a Tale of How Three Procedures Came to Be: A Letter to the Next Generations of Surgeons. Clin Plastic Surg 34 (2007) 331–355.
2. Prasad V, Kumar S, Pradhan H, Siddiqui R, Ali I. Bilateral sagittal split osteotomy a versatile approach for correction of facial deformity: A review literature. Natl J Maxillofac Surg 2021;12:8-12.
3. Böckmann R, Meyns J, Dik E, Kessler P. The Modifications of the Sagittal Ramus Split Osteotomy: A Literature Review. last Reconstr Surg Glob Open. 2014;2:e271.
4. Alves, N.; Deana, N. F. Morphometric study of mandibular foramen in macerated skulls to contribute to the development of sagittal split ramus osteotomy (SSRO) technique. Surg Radiol Anat, 36, n. 9, p. 839-845, Nov 2014.
5. Obwegeser HL. Surgical procedures to correct mandibular prognathism and reshaping of the Oral chin. In: Trauner R, Obwegeser H, editors. The surgical correction of mandibular prognathism and retrognathia with consideration of genioplasty. Oral Surg Oral Med Oral Pathol. 1957;10:677–89.
6. Dal Pont G. Retromolar osteotomy for the correction of prognathism. J Oral Surg Anesth Hosp Dent Serv. 1961;19:42–47.
7. Macintosh RB. Experience with the Sagittal Osteotomy of the Mandibular Ramus: A 13-Year Review. J. max.-fac. Surg. 8 (1981) 151-165.

8. O. Peleg, R. Mahmoud, A. Shuster et al., Orthognathic surgery complications: The 10-year experience of a single center, Journal of Cranio-Maxillo-Facial Surgery, 2021.
9. Damrongsirirat N et al. Complications related to orthognathic surgery: A 10-year experience in oral and maxillofacial training center. J of Cranio-Maxillofacial Surg. 2022. 50 (3):197:203.
10. Verweij, J. P.; Mensink, G.; Houppermans, P. N.; Van Merkesteyn, J. P. Angled Osteotomy Design Aimed to Influence the Lingual Fracture Line in Bilateral Sagittal Split Osteotomy: A Human Cadaveric Study. J Oral Maxillofac Surg, 73, n. 10, p. 1983-1993, Oct 2015.
11. Plooij, J.M. et al. 3D evaluation of the lingual fracture line after a bilateral sagittal split osteotomy of the mandible. Int J Oral Maxillofac Surg 2009 Dec;38(12):1244-9.
12. Kriwalsky MS et al. Risk factors for a bad split during sagittal split osteotomy. British Journal of Oral and Maxillofacial Surgery 46 (2008) 177–179.
13. Zeynalzadeh F, Shooshtari Z, Eshghpour M, Zarch SHH, Tohidi E, Samieirad S. Dal Pont vs Hunsuck: Which Technique Can Lead to a Lower Incidence of Bad Split during Bilateral Sagittal Split Osteotomy? A Triple-blind Randomized Clinical Trial. World J Plast Surg. 2021 Sep; 10(3): 25–33.
14. Lee, J. H.; Son, Y. J.; Hwang, J. H.; Baek, S. H. et al. Influence of anatomic position and intraoperative exposure of the inferior alveolar nerve on neurosensory disturbance after sagittal split ramus osteotomy: a three-dimensional computed tomography study. Oral Surg Oral Med Oral Pathol Oral Radiol, 122, n. 3, p. 300-305, Sep 2016.

15. Camargo IB, Van Sickels JE, Cunningham Jr LL, Simultaneous removal of third molars and completion of a sagittal split osteotomy: Effects of age and presence of third molars, Oral Surgery, Oral Medicine, Oral Pathology and Oral Radiology (2016).
16. Jiang N, et al. Risk factors for bad splits during sagittal split ramus osteotomy: a retrospective study of 964 cases. Br J Oral Maxillofac Surg (2021).
17. Cuschieri S. The STROBE guidelines. Saudi J Anaesth 2019; 13:31–4.
18. Smith, B. R., Rajchel, J. L., Waite, D. E., & Read, L. (1991). Mandibular ramus anatomy as it relates to the medial osteotomy of the sagittal split ramus osteotomy. Journal of Oral and Maxillofacial Surgery, 49(2), 112–116.
19. Telha E, Silva EDO, Melo Filho SMC, et al. Cone-beam computed tomography assessment of mandibular anatomical risk factors associated with bad splits during sagittal split ramus osteotomy. Int J Oral Maxillofac Surg. 2023;52:256-264. doi: 10.1016/j.ijom.2022.07.003.
20. Chrcanovic BR, Freire-Maia B. Risk factors and prevention of bad splits during sagittal split osteotomy. Oral Maxillofac Surg. 2012;16:19-27.
21. Scomparin RC, Barone S, Tedesco A, et al. Anatomical study of the lingula and its clinical implications in sagittal split ramus osteotomy. J Craniomaxillofac Surg. 2017;45:846-853.
22. De Souza Fernandes, A. C., Cardoso, P. M., Fernandes, I. S., & de Moraes, M. (2013). Anatomic Study for the Horizontal Cut of the Sagittal Split Ramus Osteotomy. Journal of Oral and Maxillofacial Surgery, 71(7), 1239–1244.

F1

Click here to access/download;Figure;FIG 1.tif

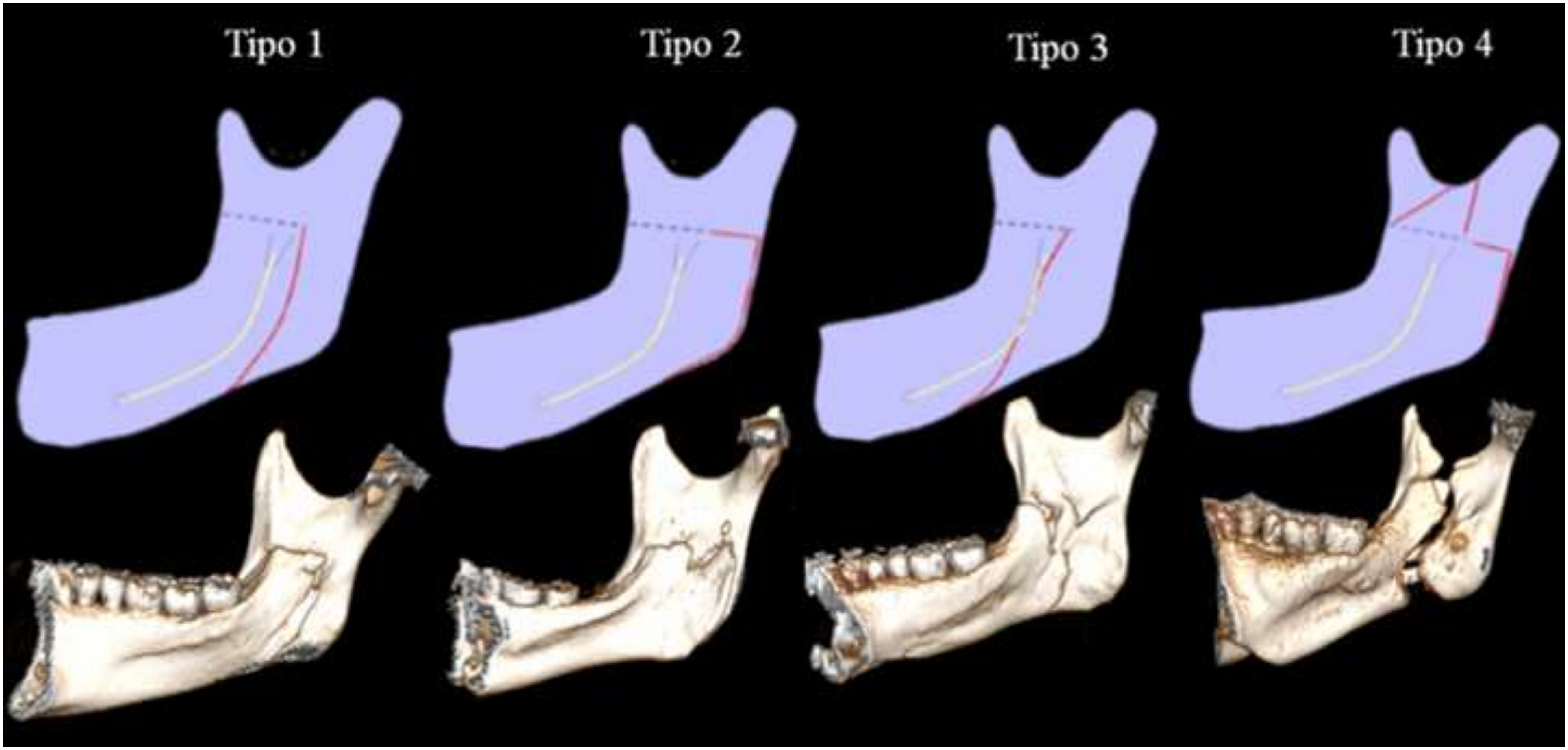

Click here to access/download;Figure;FIG 2.tif

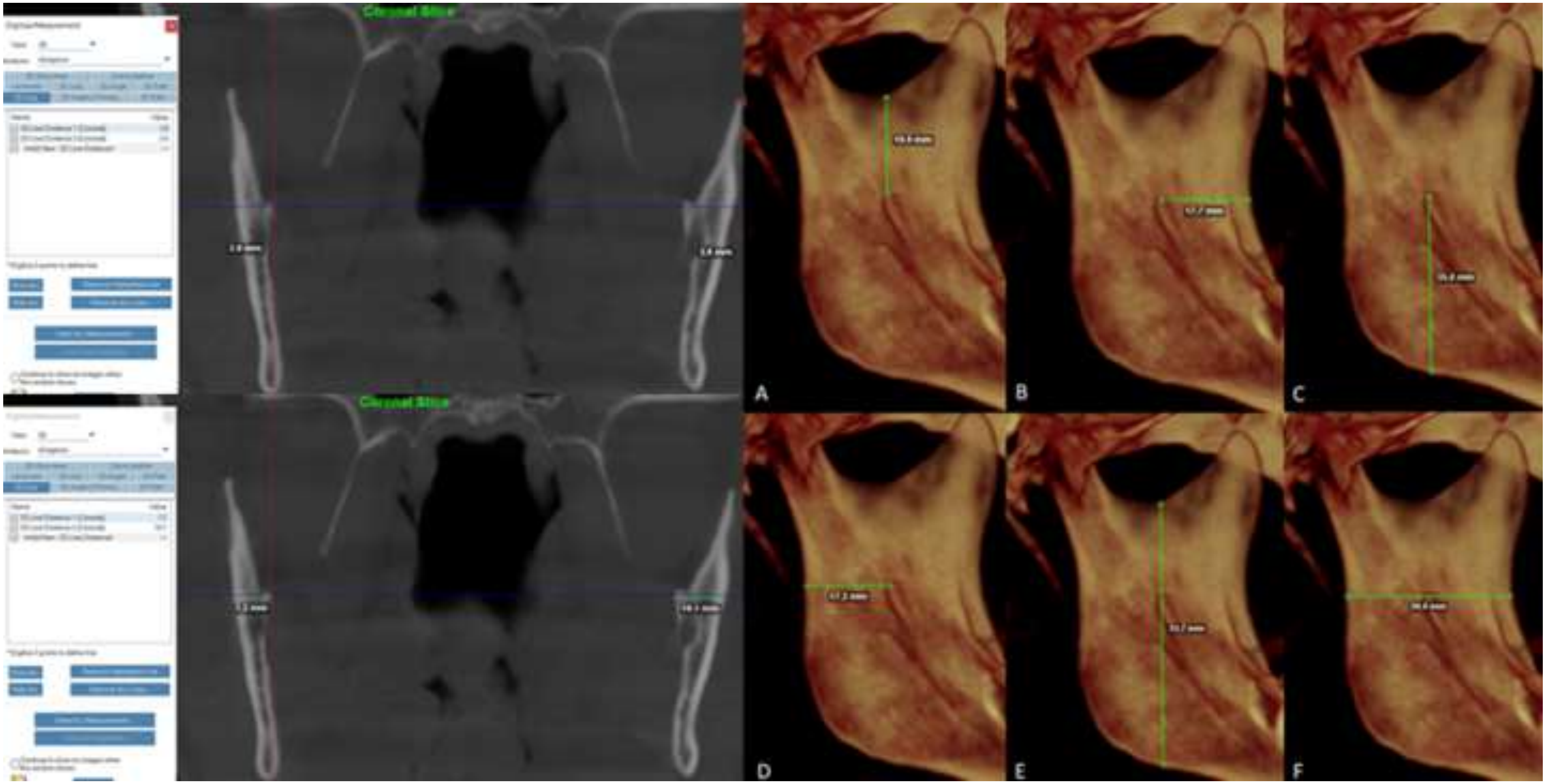

F3

Click here to access/download;Figure;FIG 3.tif

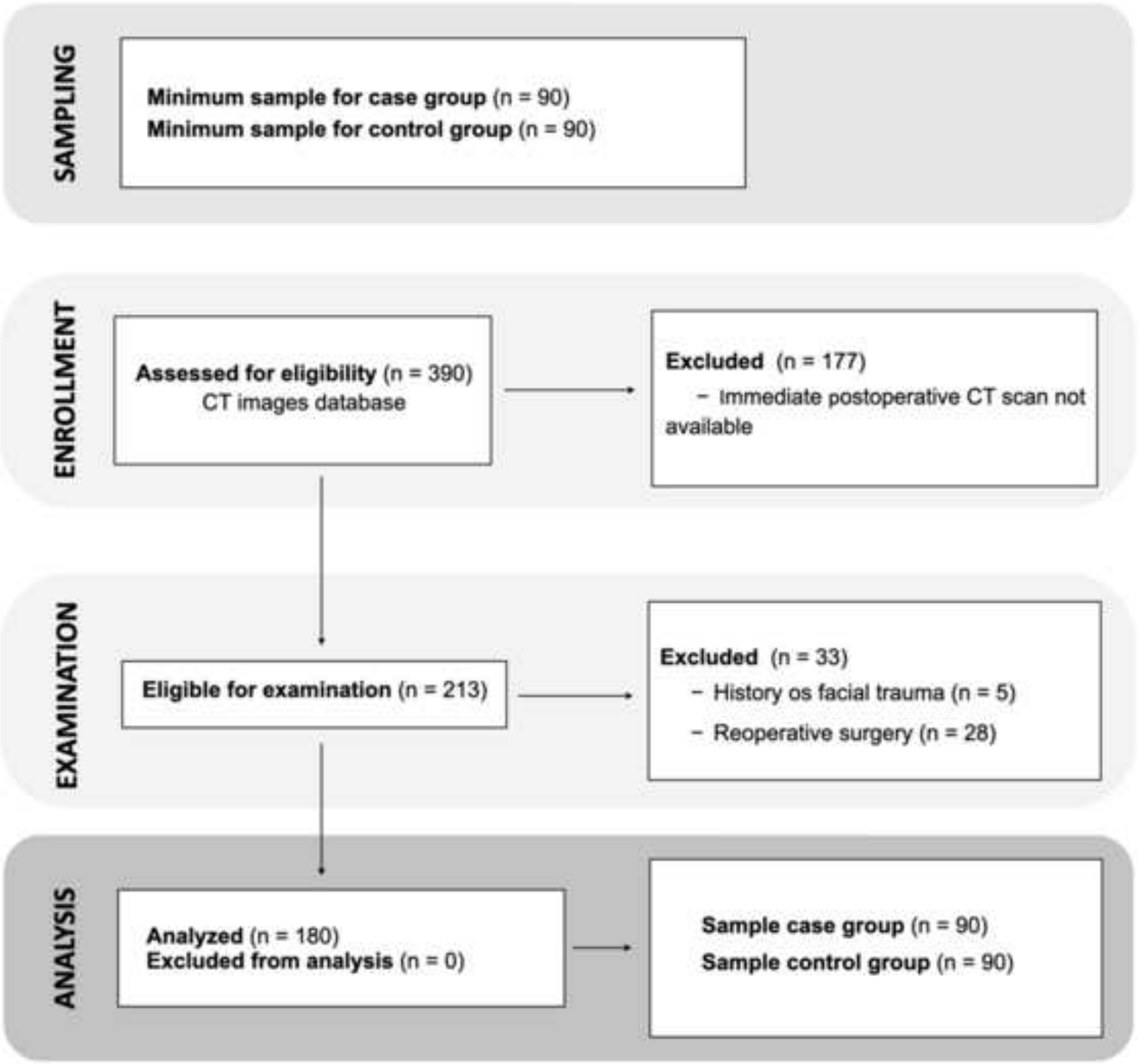

Human Participants Research Checklist

Complete the following if your study involved human participants or human participants' data. These questions should be addressed for prospective and retrospective studies.

1. Did you obtain ethics approval for this study?

   - If yes, please upload (file type "Other") the original approval document you received from your ethics committee. If the original document is in another language, please also provide an English translation.

     _X__ Uploaded ___ N/A

   - If you did not obtain ethical approval, please explain why this was not required below.

2. If you prospectively recruited human participants for the study – for example, you conducted a clinical trial, distributed questionnaires, or obtained tissues, data or samples for the purposes of this study, please report in the Methods:

   i. the day, month and year of the **start and end** of the recruitment period for this study.

   ii. whether participants provided informed consent, and if so, what type was obtained (for instance, written or verbal, and if verbal, how it was documented and witnessed). If your study included minors, state whether you obtained consent from parents or guardians. If the need for consent was waived by the ethics committee, please include this information.

   ___ Completed _X__ N/A

3. If you are reporting a retrospective study of medical records or archived samples, please report in the Methods section:

   i. the day, month and year when the data were accessed for research purposes

   ii. whether authors had access to information that could identify individual participants during or after data collection

   __X_ Completed ___ N/A

Last Modified April 2023
