# Supplementary material for: Anatomical factors associated with the lingual fracture pattern in sagittal split ramus osteotomy: A case-control study
Source: PLoS One. 2025 Aug 5;20(8):e0329542. doi: 10.1371/journal.pone.0329542 (PMC12324670; doi:10.1371/journal.pone.0329542)
Supplement: Attachment — Submitted filename: Response to Reviewers.docx [file pone.0329542.s007.docx]

Carla Cecília Lira Pereira de Castro

R. Arnóbio Marquês, 310,

Recife, Brazil 50.100-130.

E-mail: carla.castro@upe.br

July 13, 2025

To the Editorial Board of PLOS ONE.

Dear Editor,

On behalf of all the authors, I would like to thank you for the opportunity to clarify the remarks made in our manuscript entitled “*ANATOMICAL FACTORS ASSOCIATED WITH POSTERIOR FRACTURE PATTERN IN SAGITTAL SPLIT RAMUS OSTEOTOMY: A CASE-CONTROL STUDY*”. We addressed the suggestions raised by reviewers and generated the revised version of the manuscript to be considered for publication as a publication as a Review Article in this prestigious Journal.

All the changes we have made and the responses to editor and reviewer questions are listed below and highlighted in yellow in the manuscript:

**REVIEWER 1 CONSIDERATIONS**

**1. Dear Authors, Thank you for the opportunity to review your manuscript. The topic addressed is both timely and relevant, especially considering its clinical implications in orthognathic surgery. I have noted some points that may benefit from clarification or revision to strengthen the overall scientific rigor and clarity of the work. Please find below my detailed comments and suggestions for improvement.**

1. **Abstract:**

**#It omits any mention of study limitations or statistical control for intra-individual observations. Recommendation: Consider including a sentence noting the retrospective design and potential sources of bias.**

**Answer:** Thank you for your observation. Considering the retrospective design, we acknowledge the potential sources of bias inherent to the observational nature of the study. This was added to the abstract.

2. **Methods**

**#Add a brief paragraph discussing the sample size justification or power estimation**

**Answer:** Suggestion incorporated. The sample size calculation was described in the "Methods" section under the “Sample size” subsection.

**#Clarify whether bilateral rami were obtained from the same patients and, if so, apply appropriate statistical models to include the issue of intra-individual correlation, which is not accounted for in the statistical analysis.**

**Answer:** Thank you for your consideration. The mandibular rami were used as the unit of analysis given that the pattern of BSSO separation is assumed to be independent on each operated side. In some cases, the rami were obtained from the same patients. This point was clarified in the "Methods" section under “Study population and eligibility criteria” and in limitations paragraph in “Discussion” section.

3. **Results**

**#The results section does not mention how missing data (if any) were handled.**

**Answer:** There was no missing data in the study.

**#There is no clear statement about the number of patients versus number of rami, which may inflate the sample size and precision if not adjusted.**

**Answer:** As described in the "Methods" section, the mandibular ramus was considered the unit of analysis, given that both mandibular morphology and fracture patterns are independent on each side. We have clarified in the text the number of patients versus the number of rami, as suggested.

**#The tables formatting does not adhere to standard scientific style. In scientific manuscripts, tables are usually presented without visible grid lines between rows and columns.**

**Answer:** Suggestion accepted. The tables were reformatted accordingly.

4. **Discussion**

**#The authors do not address study limitations as (Retrospective design;Potential selection bias;Absence of adjustment for intra-individual correlation;No multivariate control of confounders),which is a critical omission in observational research.**

**#There is occasional language suggesting causality which should be revised to reflect associative relationships only.**

**Answer:** Study limitations were added to the “Discussion” section under the “Study Limitations” subsection.

5. **Conclusion**

**#Consider softening the conclusion by noting that the findings are associative and that further prospective studies are warranted.**

**Answer:** Suggestion implemented in conclusion.

6. **Figures and tables**

**# Figures are helpful, though Figure 1 and 2 could benefit from improved resolution and clearer anatomical labels. Consider to improve figure quality and consider labeling anatomical landmarks more explicitly for clarity.**

**#The tables formatting does not adhere to standard scientific style. In scientific manuscripts, tables are usually presented without visible grid lines between rows and columns. Please , consult PlOS One guidelines for table formating and presentation.**

**Answer:** Both suggestions were addressed. Figures were improved and tables reformatted.

**REVIEWER 2 CONSIDERATIONS**

1. **Missing reference in introduction in second alinea explaining lingual split scale (ref 11).**

**Answer:** Suggestion implemented. The reference was added.

2. **Please provide period in which these 390 cases were operated.**

**Answer:** Clarification added to the “Methods” section under “Study population and eligibility criteria” subsection. The cases were operated between January 2022 and December 2024.

3. **Are the case operated upon consecutively or were the groups selected based on the type of fracture? The incidence of the type 1 and 2 versus 3 and 4 are approximately 60% versus 40%, which suggests that there would be more controles than cases. Please explain this more extensively.**

**Answer:** The cases were not selected consecutively. They were grouped into cases and controls based on fracture type, using a 1:1 ratio. Now, we further explained the process in the "Methods" section under “Sample size” subsection.

**REVIEWER 3 CONSIDERATIONS**

1. **Could the authors include an a priori power analysis to confirm that n=90 per group was sufficient?**

**Answer:** As also suggested by Reviewer 1, a paragraph detailing the sample size calculation was added to the "Methods" section.

2. **The use of RadiAnt DICOM Viewer® for evaluating postoperative CT scans is commendable. However, the authors should consider including a brief note on the limitations of automated functions.**

**Answer:** This comment was incorporated into a paragraph of study limitations in the “Discussion” section.

3. **Figure 2 has low resolution. Please replace it with a high-resolution version.**

**Answer:** Suggestion accepted. Figure 2 was replaced with a higher-resolution version.

Thank you for your consideration!

Kind regards,

**Corresponding author:**

Carla Cecília Lira Pereira de Castro.
